# The fifth redo operation for mitral paravalvular leakage and free-floating closure device extraction: A case report^☆^

**DOI:** 10.1016/j.jtumed.2022.01.003

**Published:** 2022-02-04

**Authors:** Yasin Ozden, Yavuz Sensoz, Mehmet Eren, Osman E. Karpuzoglu, Ilyas Kayacioglu

**Affiliations:** a Department of Cardiovascular Surgery, Dr Siyami Ersek Thoracic and Cardiovascular Surgery Training and Research Hospital, Istanbul, Turkey; b Department of Cardiology, Dr Siyami Ersek Thoracic and Cardiovascular Surgery Training and Research Hospital, Istanbul, Turkey

**Keywords:** Mitral valve disease, Mitral valve disease, percutaneous intervention, Paravalvular leak, Surgery, valvular, Valvular heart disease, تسرب مجاور للصمام, مرض الصمام التاجي, التدخل عن طريق الجلد, جراحة الصمامات, مرض قلب صمامي

## Abstract

Paravalvular leakage (PVL) is a serious complication of prosthetic valve surgery. Surgical and transcatheter methods can be used for treatment. It is rare for closure devices to detach and free float in cardiac chambers. Transcatheter methods can be reused, but surgical treatment is more appropriate if this reuse is due to an increase in PVL. Here, we present a successfully operated case with a closure device freely passing through the PVL from the ventricle to the atrium, after four surgical valve replacements and two transcatheter device closures, owing to infective endocarditis.

## الملخص

يعتبر التسرب المجاور للصمام من المضاعفات الخطيرة لجراحة الصمام الاصطناعي. يمكن استخدام الطرق الجراحية وطرق القسطرة في العلاج. من النادر أن تنفصل أجهزة الإغلاق من مكانها وتتجول في غرف القلب. يمكن استخدام طرق القسطرة مرة أخرى، ولكن إذا كانت إعادة الاستخدام هذه بسبب زيادة في التسرب المجاور للصمام، فإن العلاج الجراحي يكون أكثر ملاءمة. نقدم هنا حالة تم علاجها بنجاح مع جهاز إغلاق يمر بحرية عبر التسرب المجاور للصمام من البطين إلى الأذين، بعد استبدال أربعة صمامات جراحية وإغلاق جهازين عبر القسطرة، بسبب التهاب الشغاف العدوائي.

## Introduction

Paravalvular leakage (PVL), a leak between the edge of the suture line and valvular annulus, is a complication seen in patients undergoing surgical and percutaneous transcatheter valve replacement. The frequency of PVL in mechanical valves has been reported to be 5–17%, and the total number of PVLs is increasing as a result of the gradual increase in the number of valve replacements.^1^ Significant PVLs lead to severe symptomatic heart failure and hemolytic anemia, and should be corrected. Although surgery is the gold standard for the treatment of PVL, 13%, 15%, and 35% mortality rates have been reported for the second, third, and fourth operations after the initial bioprosthetic valve replacement, respectively.^2^ Paravalvular leakage recurrence rates increase after each repeated operation.^3^ Owing to better short-term results, percutaneous transcatheter PVL closure is a technique of choice, particularly for high-risk patients.^4^ Percutaneous transcatheter closure is contraindicated in situations such as active local or systemic infection, ischemia, intracardiac thrombus, and mechanical valve instability.^5^

The patient was operated on four times for mitral valve replacement and had undergone percutaneous PVL closure two times previously. Here, we describe management of the fifth valve surgery for severe regurgitation due to large PVL with instability of the mechanical mitral valve and a free-floating closure device in the left ventricle.

## Case

A 31-year-old woman presented with congestive heart failure symptoms such as dyspnea, fatigue, peripheral edema, and venous congestion, which had gradually increased for 1 month. She had received mitral valve replacement (MVR) for infective endocarditis in 2004; was re-operated again with MVR in 2009; received aortic valve replacement and another MVR in 2015; and received a fourth MVR in 2016. All surgeries were due to infective endocarditis. After her last operation, she had an ischemic stroke, from which she recovered without any disabilities. Incipient dyspnea started in 2017, and 3D-transesophageal echocardiography revealed two PVLs, which were positioned medially at 3 o'clock and 5 o'clock according to hour plate projection, and were 9 × 4 and 3 × 4 mm, respectively. The larger leakage site was closed with an Amplatzer™ Vascular Plug III device (AVP-III) (Abbott Inc. -formerly St. Jude Medical) sized 12 × 5 mm, via a right femoral vein transseptal procedure under 3D-transesophageal echocardiography guidance. Her symptoms improved for 6 months, then increased again. A new leakage site of 10 × 4 mm at the posteromedial aspect of the valve was detected and closed with an AVP-III device (12 × 5 mm) in the same manner as in the previous intervention. The patient was symptom-free for the following 3 years.

Transthoracic echocardiography revealed normally functioning mechanical valves in aortic and mitral positions, 2 cm dehiscence of the mitral valve at the posterior commissural region, severe mitral insufficiency due to PVL, free-floating foreign body in the left ventricle, severe tricuspid insufficiency, and elevated pulmonary artery systolic pressure (PAP 100 mmHg). Fluoroscopy revealed that the object in the left ventricle was one of the misplaced closure plugs, which was freely moving through the PVL to the left ventricle and the atria (Figure 1 video). The patient was scheduled for surgery to repair the PVL of the mitral valve and remove the free-floating device.

**Figure 1:**
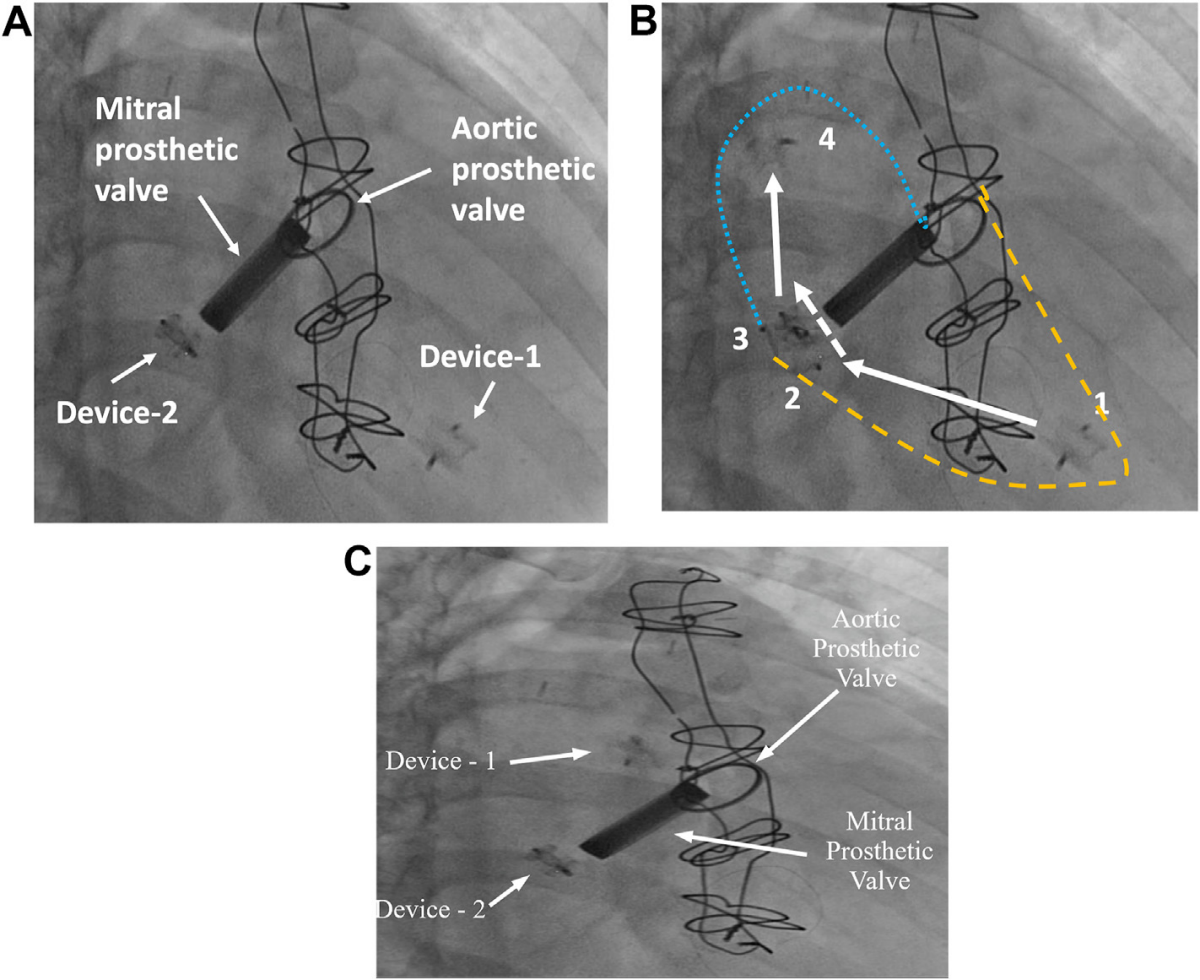
**Fluoroscopy images: A** Free-floating closure device (device 1/red circle) is in the left ventricle**. B** The direction of the movement (arrows) over time (1–4) of the free AVP-III device (device 1), observed from the left ventricle (area within the yellow cut line) toward the left atrium (area with the blue dotted line). In position 1, the device is in the left ventricle. In position 2, the device approaches the left ventricle side of the paravalvular leak with the contraction of the left ventricle. In position 3, the device, which remains in the tunnel of the paravalvular leak, passes laterally through the AVP-III device (device 2) and reaches the left atrium ceiling in position 4. **C** Device 1 (red circle) is in the left atrium.

Supplementary video related to this article can be found at https://doi.org/10.1016/j.jtumed.2022.01.003

The following is the supplementary data related to this article:

Video Free-floating closure device (device 1) in the left ventricle, observed from the left ventricle towards the left atrium. When the left ventricle contracts, device 1 passes through the tunnel laterally to the AVP-III (device 2). Video

**Figure 2:**
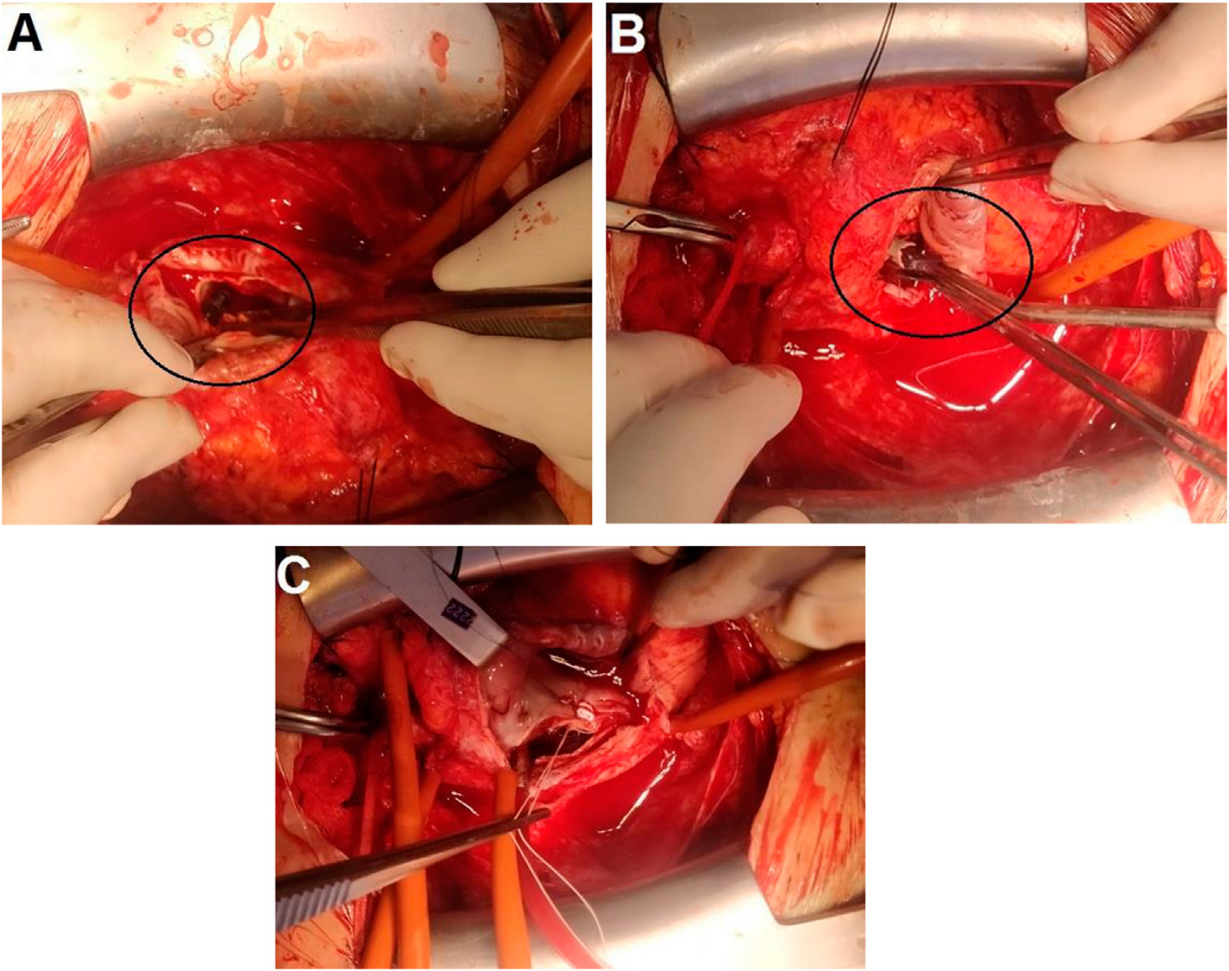
**Surgical images: A** The first AVP-III device was found free in the left atrial appendix. **B** The second AVP-III device was fitted loosely to the edge of the dehiscence slit at a 3 o'clock position from the surgical view**. C** Sutures were passed through the atrial septal tissue and annulus together to fix the sewing rim, leaving Teflon reinforcements at the atrial side.

The patient's preoperative ejection fraction was 55%; the left diastolic and systolic ventricular diameters were 59 mm and 36 mm, respectively; the interventricular septum was 11 mm; the left atrial diameter was 41 mm; and the mechanical aortic and mitral valves were functional. The left common femoral artery and vein were cannulated surgically, and the right internal jugular vein was cannulated percutaneously before sternotomy. Cardiopulmonary bypass was initiated after sternotomy to facilitate the dissection of adhesions. Cold blood cardioplegia, systemic hypothermia at 32 °C, and cross-clamping to the ascending aorta were used to arrest the heart. The left atrium was accessed via a right atriotomy and trans-septal approach. The first AVP-III device was found to be free in the left atrial appendix (Figure 2A), and the second AVP-III device was fitted loosely to the edge of the dehiscence slit, at a 3 o'clock position from the surgical view, and was removed (Figure 2B).

Because there was no sign of active endocarditis, and the paravalvular tissues were very fragile, we did not resect the mechanical valve. The mitral valve was secured tightly to the fibrous rim and septal tissue with Teflon buttressed 2-0 Ti-cron sutures. Sutures were passed through the atrial septal tissue and annulus together to fix the sewing rim, leaving Teflon strips at the atrial side (Figure 2C). Tricuspid valve annuloplasty was performed with a 34-size ring (Annuloflex, Carbomedics, Sorin Group Italia S.r.l.). Bilateral pleural effusions were drained. The patient was weaned from cardiopulmonary bypass with low-dose inotropic medication.

The ICU period was uneventful. The patient was taken to the ward on the second day and discharged on the seventh day. Postoperative transthoracic echocardiography showed functional mitral and aortic valves, no PVL, and a PAP of 40 mmHg.

Transesophageal echocardiography was performed at the first-year follow-up. No leakage was observed, and all prosthetic valves and the ring were functional.

## Discussion

Paravalvular leakage is a major complication after valve replacement operations. Surgery is the traditional method for treatment but is associated with high mortality and morbidity. For appropriate cases, transcatheter closure of PVL with percutaneous devices has lower mortality and morbidity than conventional surgery.^6^ Percutaneous closure was preferred after the fourth surgical operation in this case. Two separate sessions were required to close the leakage. In large PVLs, the defect may be closed with multiple devices. The patient's symptoms and hemolysis are expected to improve within 30 days in successful procedures. PVL is unlikely to develop until 1 year after the device closure process.^7^ Our patient remained symptom-free for approximately 3 years after the interventional procedure.

The success rate of PVL closure with a percutaneous device may be as high as 93%, and secondary interventions are rarely needed. Cruz-Gonzales et al. have reported that only 4 of 32 patients underwent a secondary procedure (three interventions and one surgical correction). The AVP-III device is the preferred device for PVL after both mitral and aortic valve surgery. The device rarely detaches and falls into the cardiac cavity because of expansion of the PVL.^8^

Secondary PVL after percutaneous closure may be associated with infective endocarditis in some situations. However, in this case, the patient's history and findings were not compatible with active infective endocarditis. Our hypothesis regarding the mechanism of PVL progression is disruption of the fragile tissue of the mitral annulus by the radial force of the AVP-III device.

The patient's heart failure symptoms began approximately 1 month before her referral, and the time when the device loosened and fell free is unknown. A free-floating device in the atria might be ensnared and removed, but echocardiographic examinations revealed that the detachment of the mechanical mitral valve at the posterior commissural area could not be closed again with closing devices, because of the instability of the valve, and therefore should be treated surgically.

Choi et al. have reported that recurrent mitral PVL after surgical correction of the PVL is not rare, and re-operation is a high-risk procedure. Repairing the PVL instead of re-replacement of the prosthetic valve would be a better surgical option, because re-replacement is an independent risk factor for PVL recurrence.^9^ We removed two closing devices, one of which was free-floating and the other of which was fixed; we repaired the detachment area, because valve re-replacement surgery would have prolonged both the duration of surgery and the cross-clamp period while the valve was functioning properly. In addition, the valvular annulus would not have been suitable for a new surgical procedure.

In patients undergoing reoperation, two surgical procedures can be performed, depending on the quality of the tissues, the size, and the location of the PVL. If tissue quality and PVL size are appropriate, repair with patches and/or Teflon reinforced sutures is preferred; otherwise, mechanical or biological valve replacement is performed. No significant difference in the type of surgical procedure (repair vs. replacement) has been observed in long-term results.^10^

Replacing the mechanical mitral valve would have increased the cross-clamp, cardiopulmonary bypass, and operation times. In addition, removal of the aortic mechanical valve might have been needed to replace the new mechanical mitral valve. Our case had a risk of posterior ventricular rupture because the mitral annulus tissue was fragile. We did not remove the valve, because the sutures in other parts of the valve were intact, and the valve was functional. The leakage area was strongly stabilized by passing the sutures with Teflon reinforcements through the mechanical valve sewing ring, the valvular annulus, and the atrial septal tissue as a whole.

If we did not find the AVP-III device in the left atrium, two options for free-floating closure device extraction in the left ventricle were possible. We could have removed the mitral and aortic mechanical valves and reached the left ventricle. Then we could have replaced both valves with new mechanical valves. If the paravalvular leakage was not large, we could have reached the device in the left ventricle through a transapical incision. We could have performed this operation through an anterior left thoracotomy, and leakage could have been closed again by transcatheter intervention. Unfortunately, the leakage was too large for transcatheter closure and might have led to a new leakage or free-floating device soon after.

We also performed an annuloplasty for the tricuspid valve and corrected the entire pathology.

Given the rarity of this case, we have described how the entire pathology was treated with the lowest risk of mortality and morbidity.

## Conclusion

Repeated valve replacement operations for infective endocarditis are ordinary situations, but the fifth consecutive operation for paravalvular leakage and extraction of a free-floating device from the ventricle made our case exceptional.

Transcatheter PVL closure may be a safe and feasible technique, but despite increasing mortality and morbidity due to multiple operations, in selected cases, surgery is the only method to resolve all problems at one time when other options are depleted.

## Source of funding

This research did not receive any specific grant from funding agencies of the public, commercial, or not-for-profit sectors.

## Conflict of interest

The author(s) have no conflict of interest to declare.

## Ethical approval

This is a retrospective case report. A study permission form was obtained from the relevant hospital (with the consent of the patient) and added to the files.

## Authors contributions

YO, ME: Drafting the work or revising it critically for important intellectual content. Yş, IK: Agreement to be accountable for all aspects of the work in ensuring that questions associated with the accuracy or integrity of any part of the work are appropriately investigated and resolved. OEK: Final approval of the version to be published. All authors have critically reviewed and approved the final draft and are responsible for the content and similarity index of the manuscript.
